# Supplementary material for: Supervised machine learning to predict smoking lapses from Ecological Momentary Assessments and sensor data: Implications for just-in-time adaptive intervention development
Source: PLOS Digit Health. 2024 Aug 23;3(8):e0000594. doi: 10.1371/journal.pdig.0000594 (PMC11343380; doi:10.1371/journal.pdig.0000594)
Supplement: S1 Table — (DOCX) [file pdig.0000594.s001.docx]

***S1 Table.*** Online screening survey.

| **What is your age (in years)?** | 0-99 |
| --- | --- |
|  |  |
| **Do you smoke cigarettes at all nowadays?** | 1) No |
|  | 2) Yes |
|  |  |
| **How many cigarettes per day do you usually smoke?** | 0-99 |
|  |  |
| **Do you live in London?** | 1) No |
|  | 2) Yes |
|  |  |
| **Are you willing to meet with a researcher at University College London twice during the 10-day study?** | 1) No |
|  | 2) Yes |
|  |  |
| **Do you own a smartphone capable of running the Fitbit and m-Path apps (i.e., Android 8.0 and up; iOS 14.0 and up)?** | 1) No |
|  | 2) Yes |
|  |  |
| **Do you have internet/Wi-Fi access for the duration of the study?** | 1) No |
|  | 2) Yes |
|  |  |
|  |  |
| **Do you smoke cigarettes at all nowadays?** | 1) No |
|  | 2) Yes |
|  |  |
| **Are you willing to set a quit date within 7 days from the initial study visit (and preferably the next day)?** | 1) No |
|  | 2) Yes |
|  |  |
| **Are you willing to wear a Fitbit device and respond to multiple daily surveys (taking a total of 48 minutes per day) on your smartphone for a period of 10 days?** | 1) No |
|  | 2) Yes |
|  |  |
| **Are you able and willing to provide an exhaled carbon monoxide (eCO) measure? Please be aware that individuals with asthma or COPD may find it difficult to provide an eCO measure.** | 1) No |
|  | 2) Yes |
|  |  |
|  |  |
| **Do you have a known history of arrythmias (e.g., atrial fibrillation)?** | 1) No |
|  | 2) Yes |
|  |  |
| **Do you regularly take beta blockers (e.g., atenolol, bisoprolol)?** | 1) No |
|  | 2) Yes |
|  |  |
| **Do you have an implanted cardiac rhythm device?** | 1) No |
|  | 2) Yes |
|  |  |
