# Supplementary material for: Supervised machine learning to predict smoking lapses from Ecological Momentary Assessments and sensor data: Implications for just-in-time adaptive intervention development
Source: PLOS Digit Health. 2024 Aug 23;3(8):e0000594. doi: 10.1371/journal.pdig.0000594 (PMC11343380; doi:10.1371/journal.pdig.0000594)
Supplement: S2 Table — (DOCX) [file pdig.0000594.s002.docx]

***S2 Table.*** Additional baseline survey questions for eligible participants.

| **What is your name?** | Free text |
| --- | --- |
|  |  |
| **What is your e-mail address?** | Free text |
|  |  |
| **What is your mobile phone number?** | Free text |
|  |  |
| **Which of the following describes how you think of yourself?** | 1) Male |
|  | 2) Female |
|  | 3) In another way  4) Prefer not to say |
|  |  |
| **What type of job do you have?** | 1) Manual |
|  | 2) Non-manual |
|  | 3) Other (e.g., student, unemployed, retired) |
|  |  |
| **What is your ethnic group?** | 1) Asian or Asian British (any Asian background)  2) Black, Black British, Caribbean or African (any Black, Black British or Caribbean background) |
|  | 3) Mixed or multiple ethnic groups (e.g., White and Black African, White and Asian) |
|  | 4) White (any White background) |
|  | 5) Other ethnic group (e.g., Arab) |
|  |  |
| **Do you have any post-16 educational qualifications?** | 1) No |
|  | 2) Yes |
|  |  |
| **How soon after waking do you have your first cigarette?** | 1) Within 5 minutes |
|  | 2) 6-30 minutes |
|  | 3) 31-60 minutes |
|  | 4) After 60 minutes |
|  |  |
| **Which of the following best describes you?** | 1) I don't want to stop smoking |
|  | 2) I think I should stop smoking but don't really want to |
|  | 3) I want to stop smoking but haven't thought about when |
|  | 4) I really want to stop smoking but don't know when I will |
|  | 5) I want to stop smoking and hope to soon |
|  | 6) I really want to stop smoking and intend to in the next 3 months |
|  | 7) I really want to stop smoking and intend to in the next month |
|  |  |
| **Have you made a serious attempt to quit smoking in the past 12 months? By serious we mean you decided that you would try to make sure you never smoked again.** | 1) No, never |
|  | 2) Yes, but not in the past year |
|  | 3) Yes, in the past year |
|  |  |
| **Have you ever used any of the following to help you stop smoking?** | 1) Nicotine replacement product (e.g. patches/gum/inhaler) without a prescription |
|  | 2) Nicotine replacement product on prescription or given to you by a health professional |
|  | 3) Zyban (bupropion) |
|  | 4) Champix (varenicline) |
|  | 5) E-cigarette or other vaping device |
|  | 6) Attended a Stop Smoking group |
|  | 7) Attended one or more Stop Smoking one-to-one counselling/advice/support sessions |
|  | 8) Phoned a smoking helpline |
|  | 9) A book or booklet |
|  | 10) Visited a website |
|  | 11) Used an application ('app') on a handled computer (smartphone, tablet, PDA) |
|  | 12) None of these |
|  | 13) Other |
|  |  |
