# Supplementary material for: Supervised machine learning to predict smoking lapses from Ecological Momentary Assessments and sensor data: Implications for just-in-time adaptive intervention development
Source: PLOS Digit Health. 2024 Aug 23;3(8):e0000594. doi: 10.1371/journal.pdig.0000594 (PMC11343380; doi:10.1371/journal.pdig.0000594)
Supplement: S3 Table — (DOCX) [file pdig.0000594.s003.docx]

***S3 Table.*** EMA items.

| **I feel sad [negative affect]** | 0-10 |
| --- | --- |
|  |  |
| **I feel irritable [negative affect]** | 0-10 |
|  |  |
| **I feel stressed [negative affect]** | 0-10 |
|  |  |
| **I feel anxious [negative affect]** | 0-10 |
|  |  |
| **I feel bored [negative affect]** | 0-10 |
|  |  |
| **What is your bodily pain intensity right now? [pain]** | 0-10 [0 = no pain; 10 = worst pain] |
|  |  |
| **I feel calm [positive affect]** | 0-10 |
|  |  |
| **I feel contented [positive affect]** | 0-10 |
|  |  |
| **I feel happy [positive affect]** | 0-10 |
|  |  |
| **I feel excited [positive affect]** | 0-10 |
|  |  |
| **I feel enthusiastic [positive affect]** | 0-10 |
|  |  |
| **I am craving a cigarette [craving]** | 0-10 |
|  |  |
| **I feel motivated NOT to smoke [motivation]** | 0-10 |
|  |  |
| **I feel confident in my ability NOT to smoke [self-efficacy]** | 0-10 |
|  |  |
| **Who are you with?** | 1) Alone |
|  | 2) With partner/spouse |
|  | 3) With friend(s) |
|  | 4) With child(ren)  5) With relative(s) |
|  | 6) With colleague(s) |
|  | 7) With stranger(s) |
|  | 8) Other |
|  |  |
| **What are you doing?** | 1) Eating/drinking |
|  | 2) Watching TV  3) Listening to music  4) Reading |
|  | 5) Working/studying  6) Walking/exercising |
|  | 7) Caring for child(ren)  8) Socialising |
|  | 9) Scrolling on social media |
|  | 10) Relaxing |
|  | 11) Doing chores  12) Other |
|  |  |
| **Where are you?** | 1) At home |
|  | 2) At school/work |
|  | 3) Outside |
|  | 4) In a restaurant/café/bar |
|  | 5) In a public place (e.g., post office) |
|  | 6) On public transport |
|  | 7) In a private vehicle |
|  | 8) In others’ home |
|  | 9) Other |
|  |  |
| **Are cigarettes available to you right now?** | 1) Easily available |
|  | 2) Available with difficulty |
|  | 3) Not available |
|  |  |
| **Have you consumed any caffeine in the last hour?** | 1) No |
|  | 2) Yes |
|  |  |
| **Have you consumed any alcohol in the last hour?** | 1) No |
|  | 2) Yes |
|  |  |
| **Have you used a nicotine product (e.g., e-cigarette, nicotine gum) in the last hour?** | 1) No |
|  | 2) Yes |
|  |  |
| **Have you smoked (even a puff) in the last hour?** | 1) No |
|  | 2) Yes |
|  |  |
| **Participant-specific variable 1** | 1) No |
|  | 2) Yes |
|  |  |
| **Participant-specific variable 2** | 1) No |
|  | 2) Yes |
|  |  |
| **Participant-specific variable 3** | 1) No |
|  | 2) Yes |
|  |  |
