# Supplementary material for: Supervised machine learning to predict smoking lapses from Ecological Momentary Assessments and sensor data: Implications for just-in-time adaptive intervention development
Source: PLOS Digit Health. 2024 Aug 23;3(8):e0000594. doi: 10.1371/journal.pdig.0000594 (PMC11343380; doi:10.1371/journal.pdig.0000594)
Supplement: S4 Table — (DOCX) [file pdig.0000594.s004.docx]

***S4 Table.*** Model performance for the four algorithm types trained and tested on the nine different predictor distance-time window combinations (with sensor data).

| **.metric** | **.estimator** | **.estimate** | **.config** | **model** | **ci_lower** | **ci_upper** | **df** |
| --- | --- | --- | --- | --- | --- | --- | --- |
| sens | binary | 0.8032787 | Preprocessor1_Model1 | rf |  |  | pd1_tw1 |
| spec | binary | 0.8654906 | Preprocessor1_Model1 | rf |  |  | pd1_tw1 |
| accuracy | binary | 0.8615702 | Preprocessor1_Model1 | rf |  |  | pd1_tw1 |
| roc_auc | binary | 0.9230394 | Preprocessor1_Model1 | rf | 0.8865803 | 0.9594985 | pd1_tw1 |
| sens | binary | 0.7213115 | Preprocessor1_Model1 | svm |  |  | pd1_tw1 |
| spec | binary | 0.8103638 | Preprocessor1_Model1 | svm |  |  | pd1_tw1 |
| accuracy | binary | 0.8047521 | Preprocessor1_Model1 | svm |  |  | pd1_tw1 |
| roc_auc | binary | 0.8080865 | Preprocessor1_Model1 | svm | 0.7373598 | 0.8788131 | pd1_tw1 |
| sens | binary | 0.7377049 | Preprocessor1_Model1 | elnet |  |  | pd1_tw1 |
| spec | binary | 0.8776185 | Preprocessor1_Model1 | elnet |  |  | pd1_tw1 |
| accuracy | binary | 0.8688017 | Preprocessor1_Model1 | elnet |  |  | pd1_tw1 |
| roc_auc | binary | 0.8932167 | Preprocessor1_Model1 | elnet | 0.8460359 | 0.9403975 | pd1_tw1 |
| sens | binary | 0.7213115 | Preprocessor1_Model1 | xgb |  |  | pd1_tw1 |
| spec | binary | 0.9095921 | Preprocessor1_Model1 | xgb |  |  | pd1_tw1 |
| accuracy | binary | 0.8977273 | Preprocessor1_Model1 | xgb |  |  | pd1_tw1 |
| roc_auc | binary | 0.9066098 | Preprocessor1_Model1 | xgb | 0.8639214 | 0.9492982 | pd1_tw1 |
| sens | binary | 0.8461538 | Preprocessor1_Model1 | rf |  |  | pd1_tw2 |
| spec | binary | 0.8604651 | Preprocessor1_Model1 | rf |  |  | pd1_tw2 |
| accuracy | binary | 0.8595041 | Preprocessor1_Model1 | rf |  |  | pd1_tw2 |
| roc_auc | binary | 0.9311015 | Preprocessor1_Model1 | rf | 0.9009799 | 0.9612230 | pd1_tw2 |
| sens | binary | 0.7692308 | Preprocessor1_Model1 | svm |  |  | pd1_tw2 |
| spec | binary | 0.7641196 | Preprocessor1_Model1 | svm |  |  | pd1_tw2 |
| accuracy | binary | 0.7644628 | Preprocessor1_Model1 | svm |  |  | pd1_tw2 |
| roc_auc | binary | 0.8466990 | Preprocessor1_Model1 | svm | 0.7952931 | 0.8981049 | pd1_tw2 |
| sens | binary | 0.8153846 | Preprocessor1_Model1 | elnet |  |  | pd1_tw2 |
| spec | binary | 0.8349945 | Preprocessor1_Model1 | elnet |  |  | pd1_tw2 |
| accuracy | binary | 0.8336777 | Preprocessor1_Model1 | elnet |  |  | pd1_tw2 |
| roc_auc | binary | 0.9060908 | Preprocessor1_Model1 | elnet | 0.8639690 | 0.9482126 | pd1_tw2 |
| sens | binary | 0.8615385 | Preprocessor1_Model1 | xgb |  |  | pd1_tw2 |
| spec | binary | 0.8194906 | Preprocessor1_Model1 | xgb |  |  | pd1_tw2 |
| accuracy | binary | 0.8223140 | Preprocessor1_Model1 | xgb |  |  | pd1_tw2 |
| roc_auc | binary | 0.9008263 | Preprocessor1_Model1 | xgb | 0.8537325 | 0.9479201 | pd1_tw2 |
| sens | binary | 0.8363636 | Preprocessor1_Model1 | rf |  |  | pd1_tw3 |
| spec | binary | 0.9025192 | Preprocessor1_Model1 | rf |  |  | pd1_tw3 |
| accuracy | binary | 0.8987603 | Preprocessor1_Model1 | rf |  |  | pd1_tw3 |
| roc_auc | binary | 0.9516081 | Preprocessor1_Model1 | rf | 0.9329187 | 0.9702974 | pd1_tw3 |
| sens | binary | 0.7454545 | Preprocessor1_Model1 | svm |  |  | pd1_tw3 |
| spec | binary | 0.7798467 | Preprocessor1_Model1 | svm |  |  | pd1_tw3 |
| accuracy | binary | 0.7778926 | Preprocessor1_Model1 | svm |  |  | pd1_tw3 |
| roc_auc | binary | 0.8654386 | Preprocessor1_Model1 | svm | 0.8219871 | 0.9088901 | pd1_tw3 |
| sens | binary | 0.8727273 | Preprocessor1_Model1 | elnet |  |  | pd1_tw3 |
| spec | binary | 0.8587076 | Preprocessor1_Model1 | elnet |  |  | pd1_tw3 |
| accuracy | binary | 0.8595041 | Preprocessor1_Model1 | elnet |  |  | pd1_tw3 |
| roc_auc | binary | 0.9435627 | Preprocessor1_Model1 | elnet | 0.9207909 | 0.9663345 | pd1_tw3 |
| sens | binary | 0.8000000 | Preprocessor1_Model1 | xgb |  |  | pd1_tw3 |
| spec | binary | 0.8619934 | Preprocessor1_Model1 | xgb |  |  | pd1_tw3 |
| accuracy | binary | 0.8584711 | Preprocessor1_Model1 | xgb |  |  | pd1_tw3 |
| roc_auc | binary | 0.9329782 | Preprocessor1_Model1 | xgb | 0.9070325 | 0.9589239 | pd1_tw3 |
| sens | binary | 0.7796610 | Preprocessor1_Model1 | rf |  |  | pd2_tw1 |
| spec | binary | 0.8668867 | Preprocessor1_Model1 | rf |  |  | pd2_tw1 |
| accuracy | binary | 0.8615702 | Preprocessor1_Model1 | rf |  |  | pd2_tw1 |
| roc_auc | binary | 0.9033208 | Preprocessor1_Model1 | rf | 0.8592480 | 0.9473937 | pd2_tw1 |
| sens | binary | 0.5932203 | Preprocessor1_Model1 | svm |  |  | pd2_tw1 |
| spec | binary | 0.8822882 | Preprocessor1_Model1 | svm |  |  | pd2_tw1 |
| accuracy | binary | 0.8646694 | Preprocessor1_Model1 | svm |  |  | pd2_tw1 |
| roc_auc | binary | 0.8139322 | Preprocessor1_Model1 | svm | 0.7519199 | 0.8759445 | pd2_tw1 |
| sens | binary | 0.7627119 | Preprocessor1_Model1 | elnet |  |  | pd2_tw1 |
| spec | binary | 0.8404840 | Preprocessor1_Model1 | elnet |  |  | pd2_tw1 |
| accuracy | binary | 0.8357438 | Preprocessor1_Model1 | elnet |  |  | pd2_tw1 |
| roc_auc | binary | 0.8907348 | Preprocessor1_Model1 | elnet | 0.8447519 | 0.9367178 | pd2_tw1 |
| sens | binary | 0.7627119 | Preprocessor1_Model1 | xgb |  |  | pd2_tw1 |
| spec | binary | 0.8525853 | Preprocessor1_Model1 | xgb |  |  | pd2_tw1 |
| accuracy | binary | 0.8471074 | Preprocessor1_Model1 | xgb |  |  | pd2_tw1 |
| roc_auc | binary | 0.8862785 | Preprocessor1_Model1 | xgb | 0.8372245 | 0.9353324 | pd2_tw1 |
| sens | binary | 0.8000000 | Preprocessor1_Model1 | rf |  |  | pd2_tw2 |
| spec | binary | 0.8914729 | Preprocessor1_Model1 | rf |  |  | pd2_tw2 |
| accuracy | binary | 0.8853306 | Preprocessor1_Model1 | rf |  |  | pd2_tw2 |
| roc_auc | binary | 0.9101286 | Preprocessor1_Model1 | rf | 0.8636886 | 0.9565686 | pd2_tw2 |
| sens | binary | 0.6769231 | Preprocessor1_Model1 | svm |  |  | pd2_tw2 |
| spec | binary | 0.7021041 | Preprocessor1_Model1 | svm |  |  | pd2_tw2 |
| accuracy | binary | 0.7004132 | Preprocessor1_Model1 | svm |  |  | pd2_tw2 |
| roc_auc | binary | 0.7777494 | Preprocessor1_Model1 | svm | 0.7105669 | 0.8449319 | pd2_tw2 |
| sens | binary | 0.8307692 | Preprocessor1_Model1 | elnet |  |  | pd2_tw2 |
| spec | binary | 0.8427464 | Preprocessor1_Model1 | elnet |  |  | pd2_tw2 |
| accuracy | binary | 0.8419421 | Preprocessor1_Model1 | elnet |  |  | pd2_tw2 |
| roc_auc | binary | 0.9038249 | Preprocessor1_Model1 | elnet | 0.8601338 | 0.9475160 | pd2_tw2 |
| sens | binary | 0.7538462 | Preprocessor1_Model1 | xgb |  |  | pd2_tw2 |
| spec | binary | 0.8449612 | Preprocessor1_Model1 | xgb |  |  | pd2_tw2 |
| accuracy | binary | 0.8388430 | Preprocessor1_Model1 | xgb |  |  | pd2_tw2 |
| roc_auc | binary | 0.8896328 | Preprocessor1_Model1 | xgb | 0.8438935 | 0.9353722 | pd2_tw2 |
| sens | binary | 0.8000000 | Preprocessor1_Model1 | rf |  |  | pd2_tw3 |
| spec | binary | 0.8964758 | Preprocessor1_Model1 | rf |  |  | pd2_tw3 |
| accuracy | binary | 0.8904959 | Preprocessor1_Model1 | rf |  |  | pd2_tw3 |
| roc_auc | binary | 0.9267438 | Preprocessor1_Model1 | rf | 0.8878608 | 0.9656267 | pd2_tw3 |
| sens | binary | 0.7166667 | Preprocessor1_Model1 | svm |  |  | pd2_tw3 |
| spec | binary | 0.8414097 | Preprocessor1_Model1 | svm |  |  | pd2_tw3 |
| accuracy | binary | 0.8336777 | Preprocessor1_Model1 | svm |  |  | pd2_tw3 |
| roc_auc | binary | 0.8459618 | Preprocessor1_Model1 | svm | 0.7828670 | 0.9090566 | pd2_tw3 |
| sens | binary | 0.8000000 | Preprocessor1_Model1 | elnet |  |  | pd2_tw3 |
| spec | binary | 0.8766520 | Preprocessor1_Model1 | elnet |  |  | pd2_tw3 |
| accuracy | binary | 0.8719008 | Preprocessor1_Model1 | elnet |  |  | pd2_tw3 |
| roc_auc | binary | 0.8749449 | Preprocessor1_Model1 | elnet | 0.8142918 | 0.9355980 | pd2_tw3 |
| sens | binary | 0.7000000 | Preprocessor1_Model1 | xgb |  |  | pd2_tw3 |
| spec | binary | 0.9284141 | Preprocessor1_Model1 | xgb |  |  | pd2_tw3 |
| accuracy | binary | 0.9142562 | Preprocessor1_Model1 | xgb |  |  | pd2_tw3 |
| roc_auc | binary | 0.9045154 | Preprocessor1_Model1 | xgb | 0.8589830 | 0.9500479 | pd2_tw3 |
| sens | binary | 0.8888889 | Preprocessor1_Model1 | rf |  |  | pd3_tw1 |
| spec | binary | 0.8960613 | Preprocessor1_Model1 | rf |  |  | pd3_tw1 |
| accuracy | binary | 0.8956612 | Preprocessor1_Model1 | rf |  |  | pd3_tw1 |
| roc_auc | binary | 0.9489424 | Preprocessor1_Model1 | rf | 0.9166093 | 0.9812755 | pd3_tw1 |
| sens | binary | 0.5740741 | Preprocessor1_Model1 | svm |  |  | pd3_tw1 |
| spec | binary | 0.7242888 | Preprocessor1_Model1 | svm |  |  | pd3_tw1 |
| accuracy | binary | 0.7159091 | Preprocessor1_Model1 | svm |  |  | pd3_tw1 |
| roc_auc | binary | 0.7325553 | Preprocessor1_Model1 | svm | 0.6573882 | 0.8077225 | pd3_tw1 |
| sens | binary | 0.8518519 | Preprocessor1_Model1 | elnet |  |  | pd3_tw1 |
| spec | binary | 0.8741794 | Preprocessor1_Model1 | elnet |  |  | pd3_tw1 |
| accuracy | binary | 0.8729339 | Preprocessor1_Model1 | elnet |  |  | pd3_tw1 |
| roc_auc | binary | 0.9118243 | Preprocessor1_Model1 | elnet | 0.8610785 | 0.9625701 | pd3_tw1 |
| sens | binary | 0.8888889 | Preprocessor1_Model1 | xgb |  |  | pd3_tw1 |
| spec | binary | 0.8741794 | Preprocessor1_Model1 | xgb |  |  | pd3_tw1 |
| accuracy | binary | 0.8750000 | Preprocessor1_Model1 | xgb |  |  | pd3_tw1 |
| roc_auc | binary | 0.9373531 | Preprocessor1_Model1 | xgb | 0.8987244 | 0.9759818 | pd3_tw1 |
| sens | binary | 0.8064516 | Preprocessor1_Model1 | rf |  |  | pd3_tw2 |
| spec | binary | 0.8874172 | Preprocessor1_Model1 | rf |  |  | pd3_tw2 |
| accuracy | binary | 0.8822314 | Preprocessor1_Model1 | rf |  |  | pd3_tw2 |
| roc_auc | binary | 0.9078722 | Preprocessor1_Model1 | rf | 0.8569230 | 0.9588215 | pd3_tw2 |
| sens | binary | 0.1290323 | Preprocessor1_Model1 | svm |  |  | pd3_tw2 |
| spec | binary | 0.9834437 | Preprocessor1_Model1 | svm |  |  | pd3_tw2 |
| accuracy | binary | 0.9287190 | Preprocessor1_Model1 | svm |  |  | pd3_tw2 |
| roc_auc | binary | 0.7517090 | Preprocessor1_Model1 | svm | 0.6818620 | 0.8215560 | pd3_tw2 |
| sens | binary | 0.7419355 | Preprocessor1_Model1 | elnet |  |  | pd3_tw2 |
| spec | binary | 0.8587196 | Preprocessor1_Model1 | elnet |  |  | pd3_tw2 |
| accuracy | binary | 0.8512397 | Preprocessor1_Model1 | elnet |  |  | pd3_tw2 |
| roc_auc | binary | 0.8753739 | Preprocessor1_Model1 | elnet | 0.8218170 | 0.9289307 | pd3_tw2 |
| sens | binary | 0.8064516 | Preprocessor1_Model1 | xgb |  |  | pd3_tw2 |
| spec | binary | 0.8675497 | Preprocessor1_Model1 | xgb |  |  | pd3_tw2 |
| accuracy | binary | 0.8636364 | Preprocessor1_Model1 | xgb |  |  | pd3_tw2 |
| roc_auc | binary | 0.8869811 | Preprocessor1_Model1 | xgb | 0.8328216 | 0.9411405 | pd3_tw2 |
| sens | binary | 0.8085106 | Preprocessor1_Model1 | rf |  |  | pd3_tw3 |
| spec | binary | 0.8914224 | Preprocessor1_Model1 | rf |  |  | pd3_tw3 |
| accuracy | binary | 0.8873967 | Preprocessor1_Model1 | rf |  |  | pd3_tw3 |
| roc_auc | binary | 0.9268603 | Preprocessor1_Model1 | rf | 0.8812102 | 0.9725103 | pd3_tw3 |
| sens | binary | 0.7021277 | Preprocessor1_Model1 | svm |  |  | pd3_tw3 |
| spec | binary | 0.7057546 | Preprocessor1_Model1 | svm |  |  | pd3_tw3 |
| accuracy | binary | 0.7055785 | Preprocessor1_Model1 | svm |  |  | pd3_tw3 |
| roc_auc | binary | 0.7824520 | Preprocessor1_Model1 | svm | 0.7083672 | 0.8565368 | pd3_tw3 |
| sens | binary | 0.8085106 | Preprocessor1_Model1 | elnet |  |  | pd3_tw3 |
| spec | binary | 0.8545060 | Preprocessor1_Model1 | elnet |  |  | pd3_tw3 |
| accuracy | binary | 0.8522727 | Preprocessor1_Model1 | elnet |  |  | pd3_tw3 |
| roc_auc | binary | 0.9011482 | Preprocessor1_Model1 | elnet | 0.8514970 | 0.9507993 | pd3_tw3 |
| sens | binary | 0.7872340 | Preprocessor1_Model1 | xgb |  |  | pd3_tw3 |
| spec | binary | 0.8512486 | Preprocessor1_Model1 | xgb |  |  | pd3_tw3 |
| accuracy | binary | 0.8481405 | Preprocessor1_Model1 | xgb |  |  | pd3_tw3 |
| roc_auc | binary | 0.8988380 | Preprocessor1_Model1 | xgb | 0.8478089 | 0.9498671 | pd3_tw3 |

*Notes.* df = prediction distance-time window combination.
