# Supplementary material for: Supervised machine learning to predict smoking lapses from Ecological Momentary Assessments and sensor data: Implications for just-in-time adaptive intervention development
Source: PLOS Digit Health. 2024 Aug 23;3(8):e0000594. doi: 10.1371/journal.pdig.0000594 (PMC11343380; doi:10.1371/journal.pdig.0000594)
Supplement: S2 Fig — (DOCX) [file pdig.0000594.s006.docx]

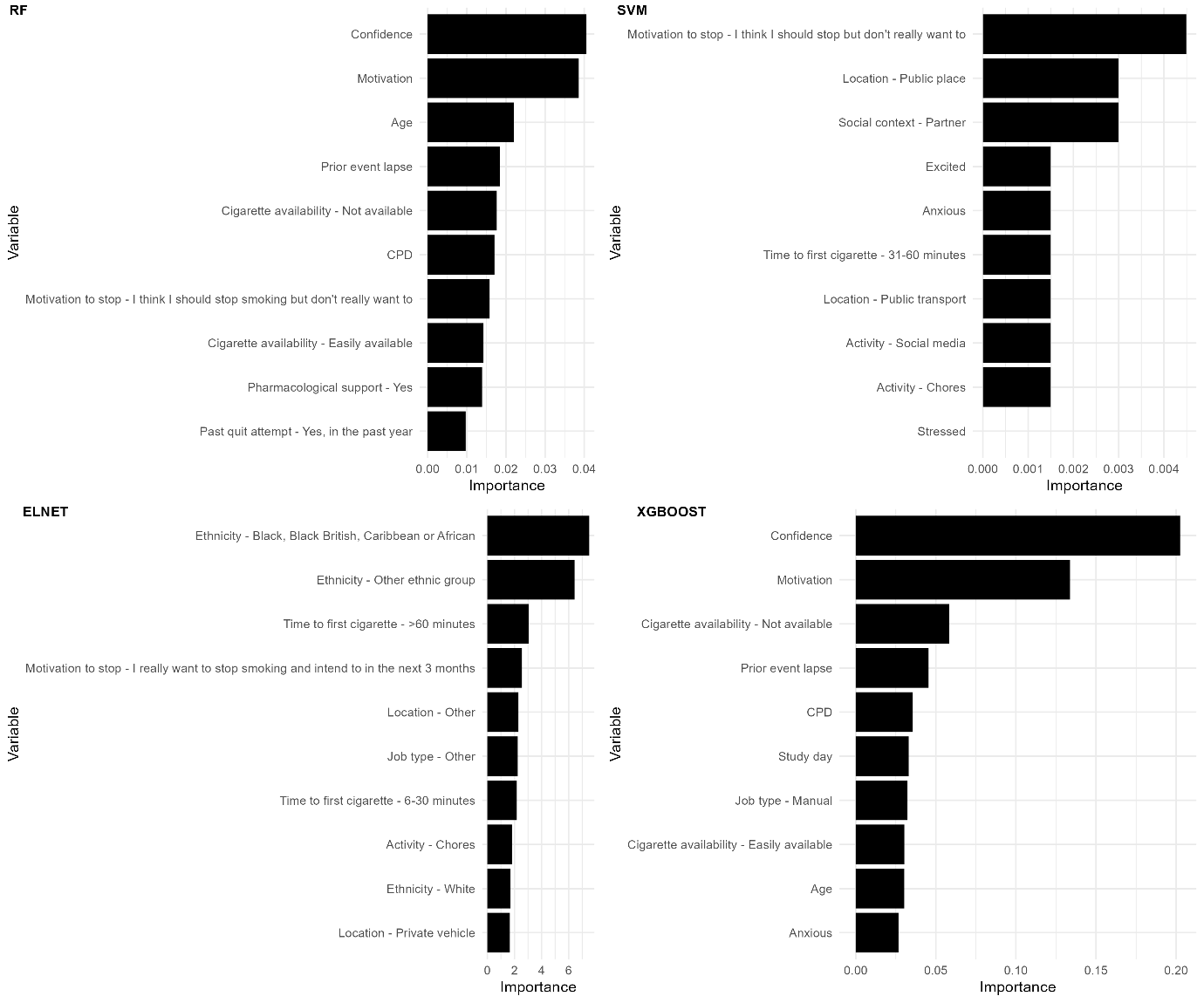


***S2 Figure.*** Variable importance plots for the best-performing group-level algorithms. The variable importance score does not indicate the direction of the relationship between the predictor and outcome variable.
