# Supplementary material for: Supervised machine learning to predict smoking lapses from Ecological Momentary Assessments and sensor data: Implications for just-in-time adaptive intervention development
Source: PLOS Digit Health. 2024 Aug 23;3(8):e0000594. doi: 10.1371/journal.pdig.0000594 (PMC11343380; doi:10.1371/journal.pdig.0000594)
Supplement: S3 Fig — (DOCX) [file pdig.0000594.s007.docx]

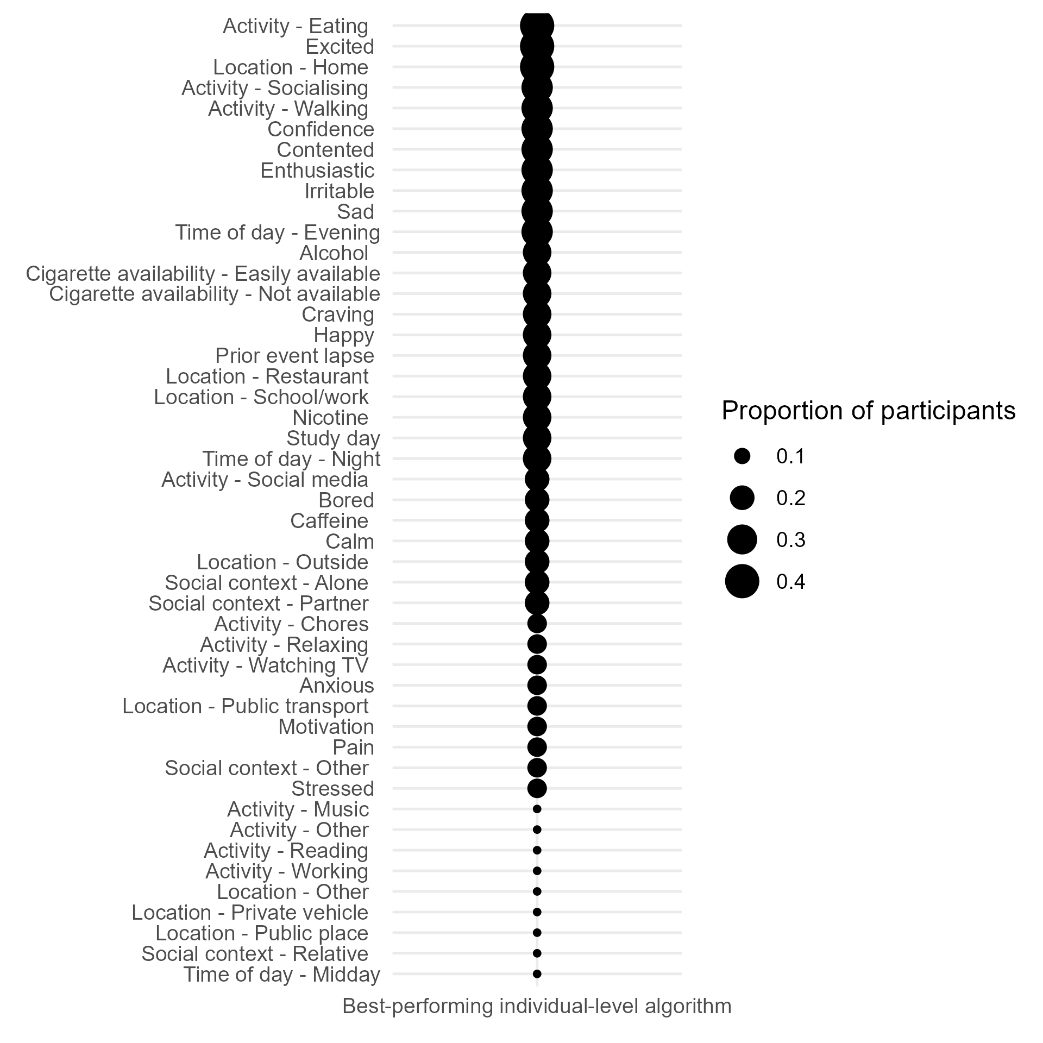


***S3 Figure.*** Proportion of participants with each of the predictor variables in their top 10 (*n* = 15). For clarity, predictor variables that were not included in a single participant’s top 10 are not displayed.
