# Supplementary material for: Supervised machine learning to predict smoking lapses from Ecological Momentary Assessments and sensor data: Implications for just-in-time adaptive intervention development
Source: PLOS Digit Health. 2024 Aug 23;3(8):e0000594. doi: 10.1371/journal.pdig.0000594 (PMC11343380; doi:10.1371/journal.pdig.0000594)
Supplement: S4 Fig — (DOCX) [file pdig.0000594.s008.docx]

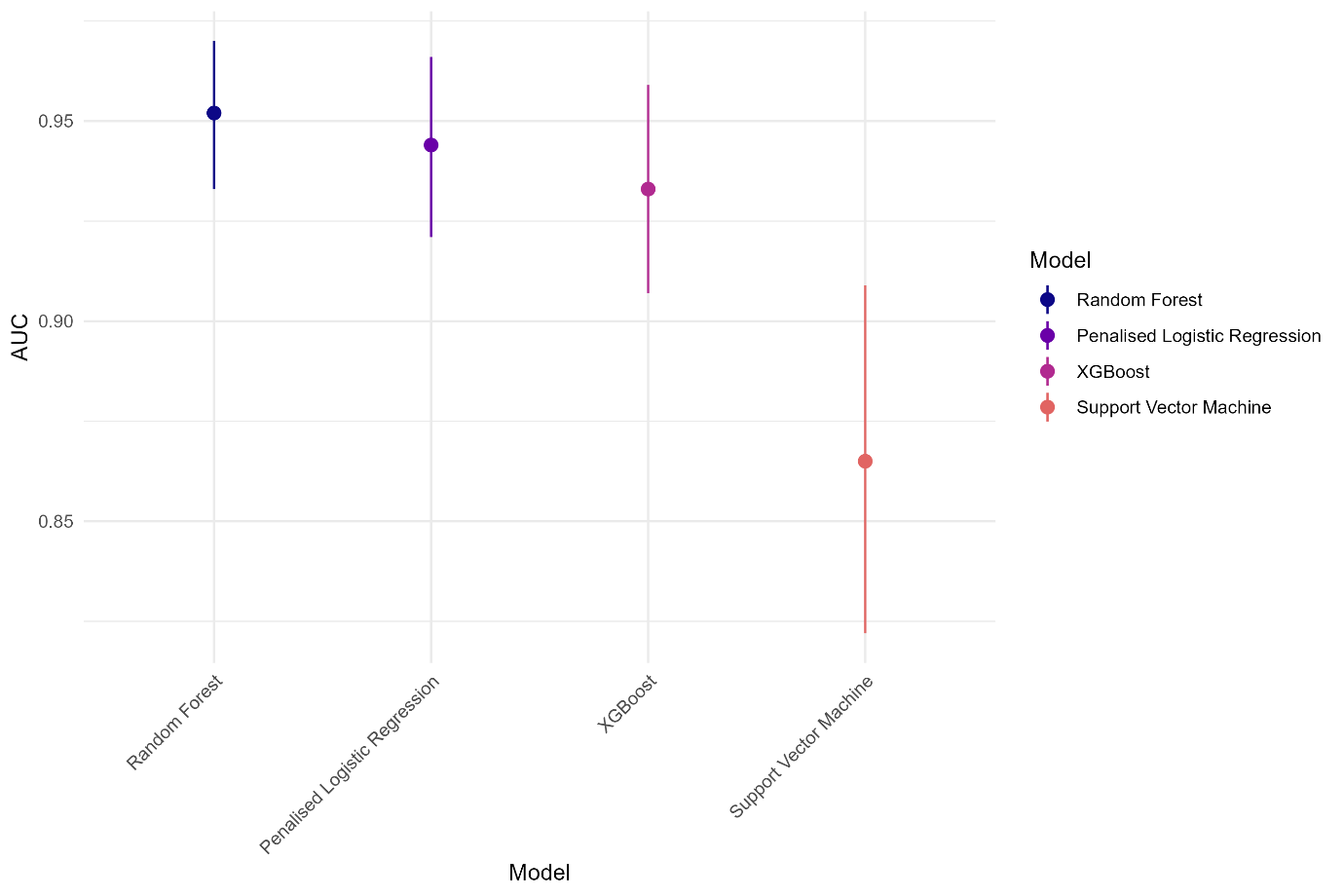


***S4 Figure.*** Area under the receiver operating characteristic curve (AUC) estimates and accompanying 95% confidence intervals for the best-performing algorithms (with sensor data).
