# Supplementary material for: Supervised machine learning to predict smoking lapses from Ecological Momentary Assessments and sensor data: Implications for just-in-time adaptive intervention development
Source: PLOS Digit Health. 2024 Aug 23;3(8):e0000594. doi: 10.1371/journal.pdig.0000594 (PMC11343380; doi:10.1371/journal.pdig.0000594)
Supplement: S7 Fig — (DOCX) [file pdig.0000594.s011.docx]

***EMA data only, with cravings as outcome***

*Objective 1 - Identifying a best-performing group-level algorithm*

The best-performing group-level algorithm was an RF algorithm (AUC = 0.918, 95% CI = 0.902 to 0.934; see S7 Figure). This was almost identical to the XGBoost algorithm, but which had a numerically lower specificity score (AUC = 0.918, 95% CI = 0.901 to 0.934). The Penalized Logistic Regression algorithm (AUC = 0.844, 95% CI = 0.821 to 0.854) and SVM algorithm (AUC = 0.830, 95% CI = 0.805 to 0.854) had worse performance.


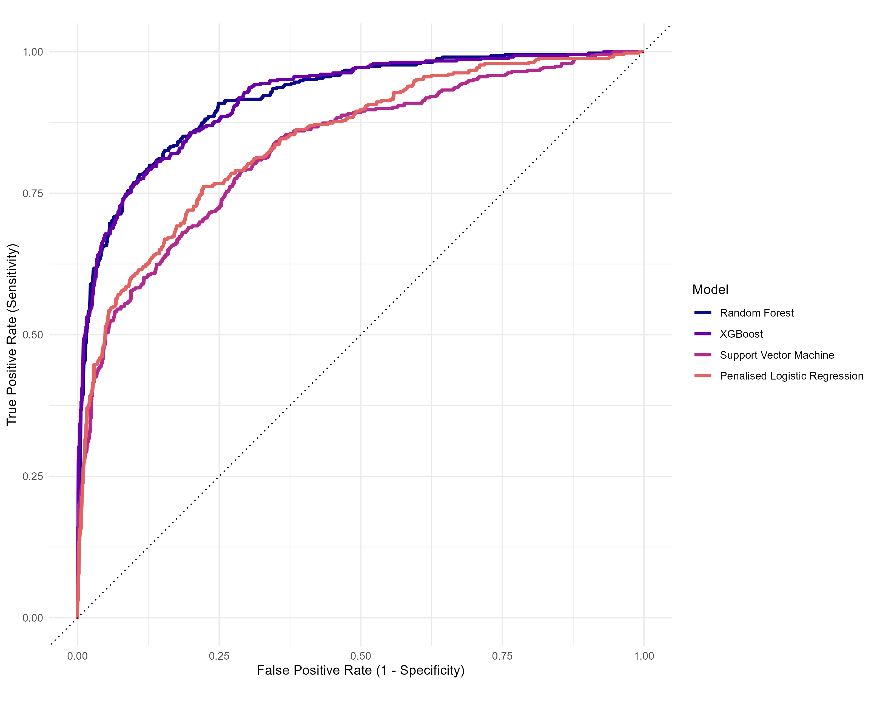


***S7 Figure.*** Plot of the area under the receiver operating characteristic curve (AUC) estimate for each of the group-level algorithms (sensitivity analysis).
