# Supplementary material for: Supervised machine learning to predict smoking lapses from Ecological Momentary Assessments and sensor data: Implications for just-in-time adaptive intervention development
Source: PLOS Digit Health. 2024 Aug 23;3(8):e0000594. doi: 10.1371/journal.pdig.0000594 (PMC11343380; doi:10.1371/journal.pdig.0000594)
Supplement: S10 Fig — The shaded grey areas represent the prespecified thresholds for acceptable accuracy (0.70), sensitivity (0.70), specificity (0.50), and AUC (0.50). The solid vertical lines represent the median. (DOCX) [file pdig.0000594.s014.docx]

*Objective 2 - Performance of the best-fitting group-level algorithms for out-of-sample individuals*

After removing participants with 0% or 100% high craving scores, algorithm performance could be computed for 37 participants (37/38; 97%). The median AUC was moderate at 0.628; however, this metric varied widely across participants (range: 0.444–0.927).

*Objective 3 - Identifying best-performing individual-level algorithms*

After removing participants with an insufficient number of high and low craving scores, algorithm performance metrics could be computed for 31 participants (31/38; 82%). S10 Figure illustrates the frequency distribution of the performance metrics of interest for participants’ best-performing algorithms. The median AUC for participants’ best-performing algorithms was 0.861 (range: 0.560 to 0.973).


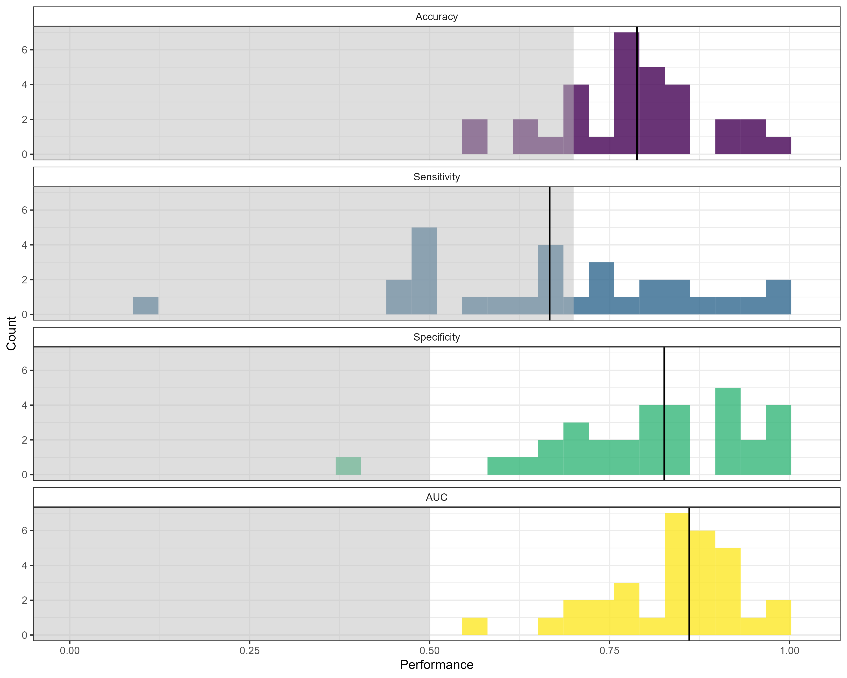


***S10 Figure.*** Frequency distributions of the performance metrics of interest (i.e., accuracy, sensitivity, specificity, AUC) for the best-performing individual-level algorithms (n = 31; sensitivity analysis). The shaded grey areas represent the prespecified thresholds for acceptable accuracy (0.70), sensitivity (0.70), specificity (0.50), and AUC (0.50). The solid vertical lines represent the median.

In an analysis examining the number of participants for whom the individual-level algorithm provided a benefit over the group-level algorithm, the individual-level algorithm was superior for most participants (28/31; 90.3%).
