# Supplementary material for: Supervised machine learning to predict smoking lapses from Ecological Momentary Assessments and sensor data: Implications for just-in-time adaptive intervention development
Source: PLOS Digit Health. 2024 Aug 23;3(8):e0000594. doi: 10.1371/journal.pdig.0000594 (PMC11343380; doi:10.1371/journal.pdig.0000594)
Supplement: S11 Fig — (DOCX) [file pdig.0000594.s015.docx]

Next, we examined the proportion of participants with each of the predictor variables in their top 10 list, estimated using the *vip* function applied to their best-performing individual-level algorithm (n = 31; see S11 Figure). For example, ‘study day’ and ‘irritable’ were included in 60% and 50% of participants’ top 10 lists, respectively.


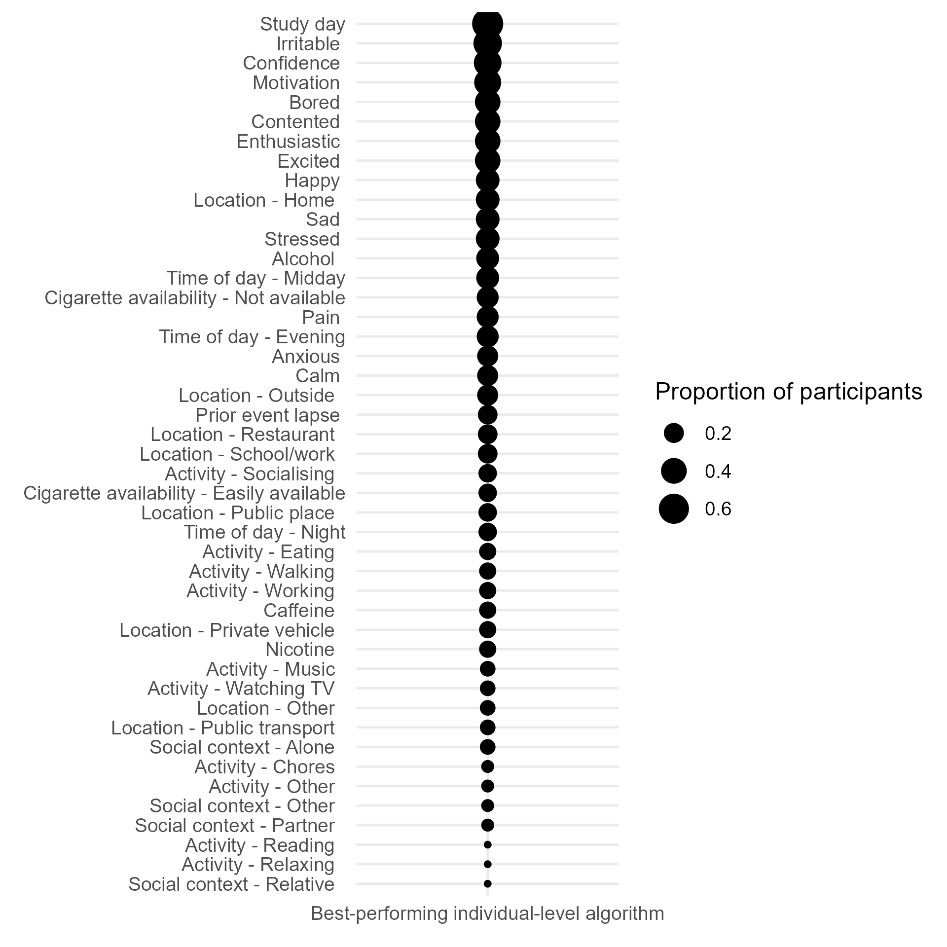


***S11 Figure.*** Proportion of participants with each of the predictor variables in their top 10 (*n* = 31; sensitivity analysis). For clarity, predictor variables that were not included in a single participant’s top 10 are not displayed.

*Objective 4 - Performance of a hybrid model for individuals*

When repeating the analyses conducted to address Objective 2 but with 20% of the individual’s data included in the training set (n = 37), the median AUC was 0.693 (range: 0.454 to 0.913). The hybrid algorithm was superior to the group-level algorithm for 65% (24/37) of participants and could be produced for 37 compared with the 31 for whom individual-only algorithms could be constructed.
