# Supplementary material for: Supervised machine learning to predict smoking lapses from Ecological Momentary Assessments and sensor data: Implications for just-in-time adaptive intervention development
Source: PLOS Digit Health. 2024 Aug 23;3(8):e0000594. doi: 10.1371/journal.pdig.0000594 (PMC11343380; doi:10.1371/journal.pdig.0000594)
Supplement: S12 Fig — (DOCX) [file pdig.0000594.s016.docx]

***EMA and sensor data, removing timepoints with potential confounders (i.e., walking/exercising, caffeine intake, nicotine use)***

*Objective 1 - Identifying a best-performing group-level algorithm*

The best-performing group-level algorithm was an RF algorithm (AUC = 0.947, 95% CI = 0.923 to 0.972; see S12 Figure), which used prediction distance 3 (45 minutes prior to the EMA prompt) and time window 1 (5 minutes of data). All subsequent analyses used this prediction distance-time window combination.


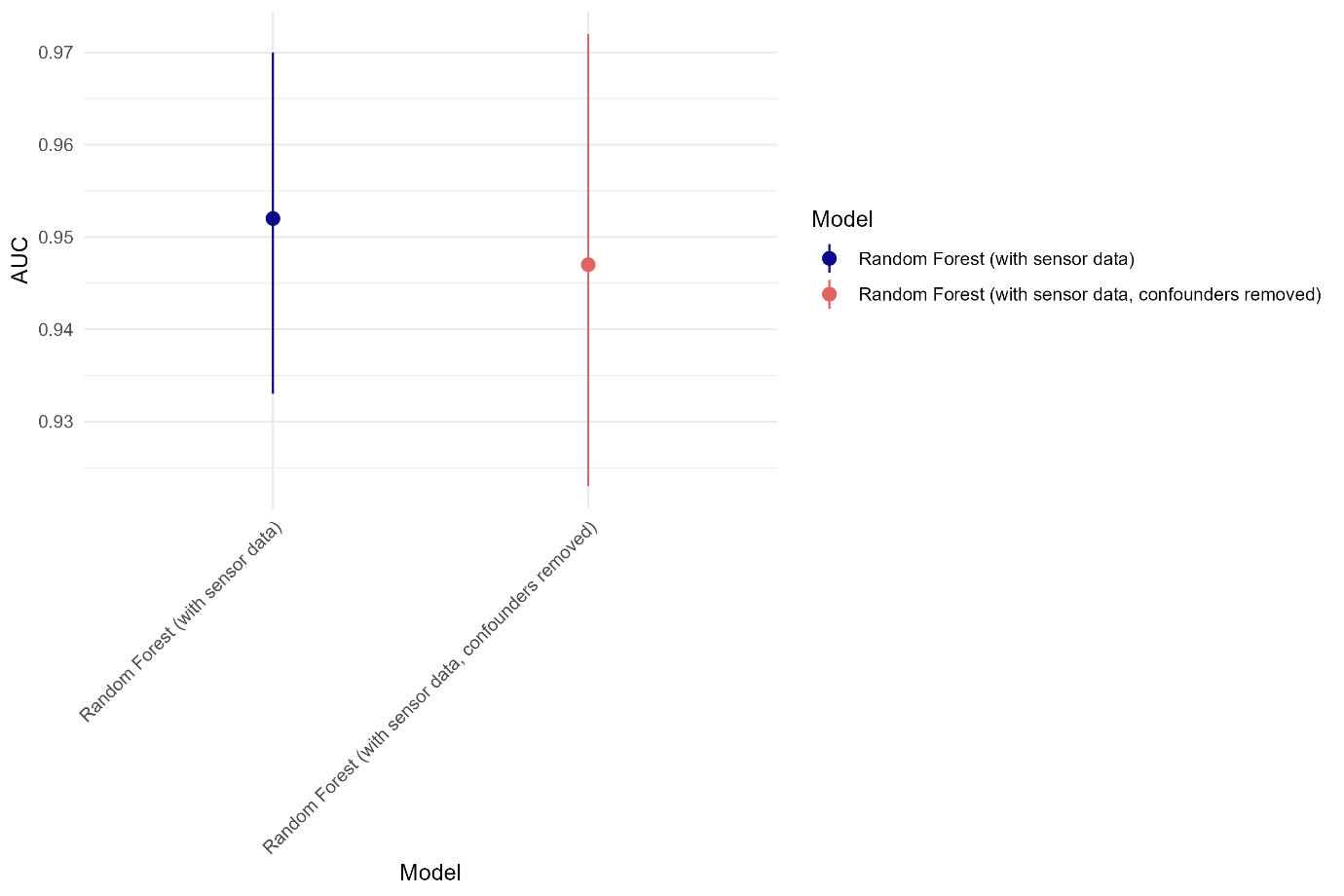


***S12 Figure.*** Area under the receiver operating characteristic curve (AUC) estimates and accompanying 95% confidence intervals for the best-performing group-level models with sensor data (without and with the removal of timepoints with potential confounders).
