# Supplementary material for: Supervised machine learning to predict smoking lapses from Ecological Momentary Assessments and sensor data: Implications for just-in-time adaptive intervention development
Source: PLOS Digit Health. 2024 Aug 23;3(8):e0000594. doi: 10.1371/journal.pdig.0000594 (PMC11343380; doi:10.1371/journal.pdig.0000594)
Supplement: S13 Fig — (DOCX) [file pdig.0000594.s017.docx]

*Objective 2 - Performance of the best-fitting group-level algorithms for out-of-sample individuals*

After removing participants with 0% lapses, algorithm performance could be computed for 17 participants (17/30; 56.7%). The median AUC was moderate at 0.737 and varied widely across participants (range: 0.545–0.988).

*Objective 3 - Identifying best-performing individual-level algorithms*

After removing participants with insufficient lapse and non-lapse events, algorithm performance metrics could be computed for 9 participants (9/30; 30%). The median AUC for participants’ best-performing algorithms was 0.909 (range: 0.606 to 1.000).

Next, we examined the proportion of participants with each of the predictor variables in their top 10 list, estimated using the *vip* function applied to their best-performing individual-level algorithm (n = 9; see S13 Figure). For example, ‘change in slope – heart rate’ and ‘location – home’ was included in 60% and 40% of participants’ top 10 lists, respectively.


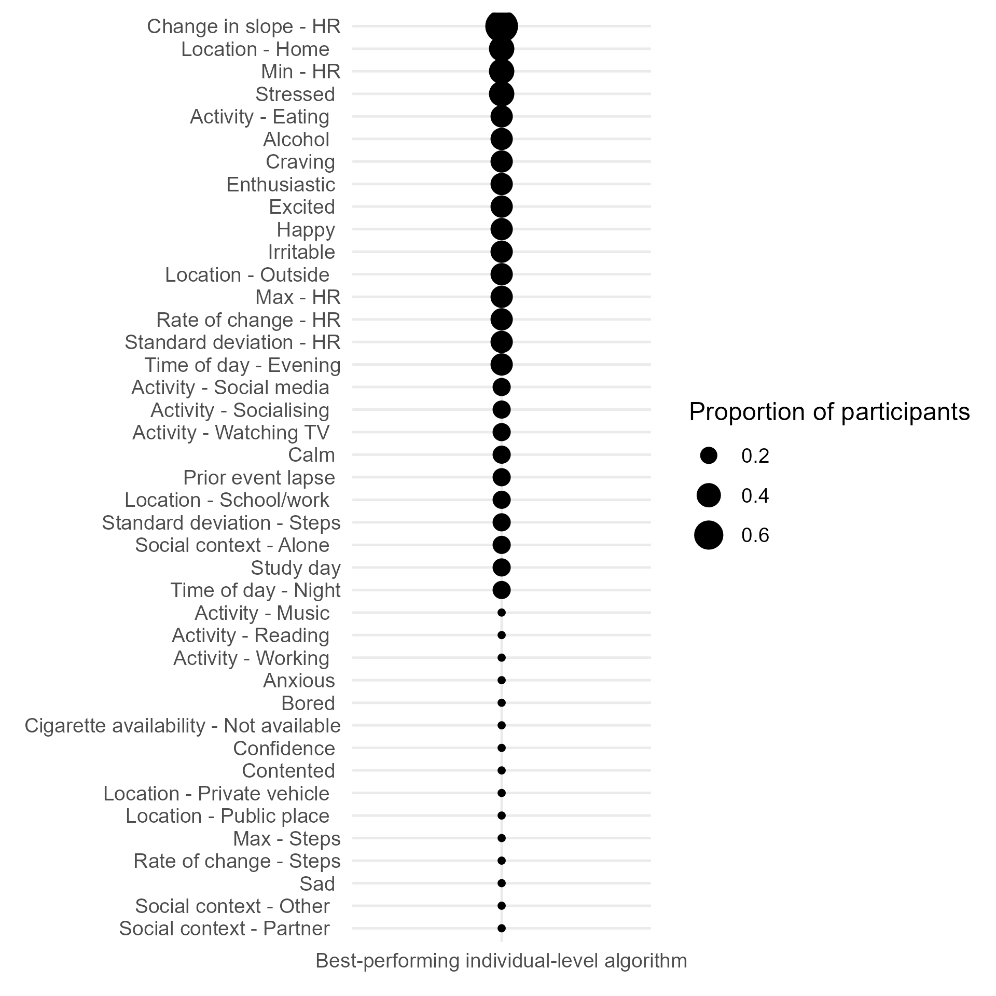


***S13 Figure.*** Proportion of participants with each of the predictor variables in their top 10 (*n* = 9). For clarity, predictor variables that were not included in a single participant’s top 10 are not displayed.

*Objective 4 - Performance of a hybrid model for individuals*

When repeating the analyses conducted to address Objective 2 but with 20% of the individual’s data included in the training set (n = 17), the median AUC was 0.744 (range: 0.457 to 1.000).
