# Supplementary material for: Supervised machine learning to predict smoking lapses from Ecological Momentary Assessments and sensor data: Implications for just-in-time adaptive intervention development
Source: PLOS Digit Health. 2024 Aug 23;3(8):e0000594. doi: 10.1371/journal.pdig.0000594 (PMC11343380; doi:10.1371/journal.pdig.0000594)
Supplement: Attachment — Submitted filename: ReviewerComments_PLOSDigitalHealth.docx [file pdig.0000594.s018.docx]

This is a comprehensive paper that does a great job of highlighting the background/significance of this study and overviewing its findings. There are some elements that can be improved to make it an excellent candidate for publication.

**Decision: Accept pending revisions**

**Main comments:**

1. The study includes an interesting combination of data sources (passive data from smartwatches and self-reported data from surveys)
2. The introduction provides a good explanation for why JITAIs are particularly relevant for smoking cessation. The authors include a great discussion of limitations of previously existing JITAIs with inclusion of specific examples
   1. An overview table summarizing key limitations of prior JITAIs compared to this one could supplement the introduction beyond the text that is included, making it clearer how this study advances on others
3. Methods include a comprehensive description of the study design. However, I do not see a description of the overall timeframe for the study – this information should be included. For instance, I wonder how seasonality / time of year might affect your results if participant data were collected in different months.
4. The inclusion of power analysis to evaluate needed sample sizes for statistical analysis is great and ameliorates my concerns about sample size for group-level analysis. However, I am concerned about sample size limitations for the individual-level analysis. The histograms in Figures 4 and 7 don’t seem very informative because the sample size is so low. Given the results, I would suggest spending include more figures/discussion for the group-level analysis and less on the individual-level analysis.
5. AUC results (Figures 2 and 5) for group-level analysis could also use a box plot or histogram (similar to Figures 4 and 7) that includes standard deviations to show how close the AUCs from the different models are to one another
6. The authors include a good discussion of using cravings vs. lapses as an outcome and warm start approaches
7. Introduction could use a few more sentences clarifying how this study is related to identifying best intervention time over best intervention method. This point is included in the Discussion, but it should be made clear earlier on.
8. Discussion could use an interpretation of the ML results – why do specific models work better than others for the various data settings?
9. Discussion could also use an interpretation of the variable importance results – why might certain variables matter more than others?
10. Discussion could use more of a description of limitations / confounders and reproducibility in the future
    1. Population applicability – It should be mentioned that current results are limited to individuals from a London-based population and need to be explored in different populations with different environments and different socioeconomic status
11. Discussion could use a description of how to translate/productionalize this work – I.e., a few more sentences about how these results might inform future JITAI development.

**Minor comments:**

1. GitHub is organized well and provides a good overview of the code for analysis. Commenting in scripts could be improved a bit further, and the README file could include a better overview of the importance of each script.
2. Small grammar error: “Finally, although this study aimed to devise a method to help researchers determine when to intervene, it not address the issue of how to intervene at moments of vulnerability”
3. Introduction: “Other approaches to the identification of JITAI decision points and tailoring variables (not expanded on further in this paper)…”
   1. This is a long sentence and is hard to follow. Break it down into smaller pieces.
4. The font size for Figures 2 to 7 could be increased to improve readability
