# Supplementary material for: Supervised machine learning to predict smoking lapses from Ecological Momentary Assessments and sensor data: Implications for just-in-time adaptive intervention development
Source: PLOS Digit Health. 2024 Aug 23;3(8):e0000594. doi: 10.1371/journal.pdig.0000594 (PMC11343380; doi:10.1371/journal.pdig.0000594)
Supplement: Attachment — Submitted filename: Responses to reviewer comments 28.03.24.docx [file pdig.0000594.s019.docx]

**Responses to reviewer comments**

*We would like to thank the three reviewers for their thoughtful comments on our manuscript, which we believe have helped improve it. Please find point-by-point responses below.*

Reviewer #1

Research Contributions

I felt the research contribution of this work seems to be slightly vague. I would also suggest the authors to include a brief contribution claim at the end of the introductory section such as the key findings, instead of listing out the objective of the study? The current manuscript, I personally feel, looks more descriptive (like a technical/UX report), instead of a paper. While I could see the insightful findings from the discussion, it would be possibly helpful to claim the key contribution at the very beginning?

*In response to this comment and similar comments from the other reviewers, we have now substantially rewritten the Introduction (edits in track changes) and included a summary table. In addition, we have clarified the key contribution of our study on p.6:*

*“The key contribution of the present study was therefore to add to the growing literature on the identification of JITAI decision points and tailoring variables by developing and evaluating a series of group-level, individual-level, and hybrid supervised machine learning algorithms to predict lapse risk in smokers attempting to stop, leveraging both EMAs and wearable sensor data. This was with a view to ultimately using the best-performing algorithm within a subsequent JITAI for smoking cessation.”*

*The key findings are also stated at the outset in the Abstract conclusions and in the Author Summary.*

Study Duration and the Participants

I am wondering if the 10 days are sufficient to verify the claimed finding, which seems somewhat short. I am also wondering if there is any reporting related to how long the recruited participants have of the smoking history?

*We have clarified the study duration on p.6:*

*“Research shows that most smokers lapse and relapse (i.e., return to regular smoking) within the first week of the quit attempt (33). We therefore opted for a high measurement frequency per day within this critical time window to capture rapid transitions from abstinence to lapse and relapse.”*

*Regarding participants’ smoking history, we collected data on (and included in the group-level algorithms) the level of nicotine dependence and past year quit attempts, which are important predictors of smoking cessation. Please see the Supplementary Materials for the survey items and response options.*

Chosen of the Algorithms

I am wondering if the authors have tried out other machine learning or deep learning algorithms? I felt a simple vanilla ANN with few number of hidden layers seems to be a nice candidate model to evaluate? I don’t think the computational overhead would be too heavy to be deployed on the edge devices.

*We appreciate that more advanced learning algorithms could be evaluated; however, we selected the four algorithm types to try based on several criteria, including their relatively good interpretability. We therefore prefer not to test additional algorithms in the present study. We have added this as a suggestion for future research on p.24:*

*“Fourth, we selected a limited number of algorithm types to try based on their relatively low computational demands, the availability of off-the-shelf R packages, and their relatively good interpretability compared with approaches such as deep learning. However, improved prediction performance may be achieved with more advanced machine learning or deep learning algorithms, such as an artificial neural network.”*

Structures, Formatting and Misc.

Page 4 – 6: it looks the introduction seems to be mixed with the related work/background section. I personally would suggest the author to see if there are any possibilities to separate them, to enhance the readability. The key focus is to justify the fundamental different to the existing related works, such as (11, 19,  23 - 26)

*In response to this comment and similar comments from the other reviewers, we have now substantially rewritten the Introduction and included a summary table.*

In the penultimate paragraph of the page 5, the format of the in-line citation seems not to be consistent. I would suggest the authors use the same numerical citation format.

*Thank you for spotting this - it has now been amended.*

Figure 1 seems to be “over-cropped” at the bottom? I would also suggest to revise the caption. It is quite vague by describing the figure as “participant flow chart”?

*We have double checked the figure and it contains the intended information. We have revised the caption to better capture the information contained: “Participant flow chart illustrating the participant screening, enrolment, and inclusion in the study analyses”.*

Font size of the Figure 2, 4, 5, and 7 possibly needs to be increased.

*We have now increased the font size in these figures.*

Reviewer #2

Major comments:

An overview table summarizing key limitations of prior JITAIs compared to this one could supplement the introduction beyond the text that is included, making it clearer how this study advances on others

*In response to this comment and similar comments from the other reviewers, we have now substantially rewritten the Introduction (edits in track changes) and included a summary table. In addition, we have clarified the key contribution of our study on p.6:*

*“The key contribution of the present study was therefore to add to the growing literature on the identification of JITAI decision points and tailoring variables by developing and evaluating a series of group-level, individual-level, and hybrid supervised machine learning algorithms to predict lapse risk in smokers attempting to stop, leveraging both EMAs and wearable sensor data. This was with a view to ultimately using the best-performing algorithm within a subsequent JITAI for smoking cessation.”*

Methods include a comprehensive description of the study design. However, I do not see a description of the overall timeframe for the study – this information should be included. For instance, I wonder how seasonality / time of year might affect your results if participant data were collected in different months.

*Thank you for spotting this omission – we have now added the following to the Methods on p.6:*

*“This was a 10-day intensive, longitudinal, observational study with participants recruited between May 2022 and March 2023.”*

*We have added the following to the limitations on p.23:*

*“Finally, participants were recruited during different months of the year. It is possible that seasonality might have affected the results. In addition, the current results are limited to London-based individuals who were willing to participate in a research study with high measurement frequency. External algorithm validation in different populations (e.g., individuals with lower socioeconomic position) and settings (e.g., individuals residing in rural or smaller urban environments) is therefore needed. We encourage such explorations, including examinations of seasonality, using our openly available R code in future research.”*

The inclusion of power analysis to evaluate needed sample sizes for statistical analysis is great and ameliorates my concerns about sample size for group-level analysis. However, I am concerned about sample size limitations for the individual-level analysis. The histograms in Figures 4 and 7 don’t seem very informative because the sample size is so low. Given the results, I would suggest spending include more figures/discussion for the group-level analysis and less on the individual-level analysis.

*We agree that the individual-level analyses need to be interpreted with caution since they could only be constructed for a small number of participants, which was primarily a consequence of the distribution of the outcome variable of interest (i.e., imbalances between lapse and non-lapse events) and our pre-registered selection of performance metrics (highlighted in the Discussion on p.23):*

*“The selected performance metrics (i.e., sensitivity, specificity, the area under the receiver operator characteristic curve) cannot be computed when the test set contains only one of the two classes. This was the case for a non-trivial proportion of participants who reported 0% lapses and hence limited the number of participants for whom the individual-level and hybrid algorithms could be constructed. Future research should consider using the Matthews Correlation Coefficient for imbalanced datasets (70), in addition to devising appropriate cut-offs for use in the context of a smoking lapse estimator, which are currently lacking.”*

*However, the within-person sample size was larger than in similar published studies and in line with larger sample sizes evaluated in simulation studies, which lends confidence to the individual-level algorithms that could be constructed. Since the group-level algorithms had highly variable performance when applied to out-of-sample individuals, we believe it is important not to overemphasise their utility. We have therefore opted to keep the results section as is and we have instead added more discussion about the methodological challenges encountered on p.23 and emphasised that the individual-level algorithms could only be constructed for a limited number of participants in the Discussion on p.20 and, the Conclusion on p.25, and in the Abstract conclusion:*

*“Related to this, research shows that supervised algorithm performance is impacted by several factors, including the sample size, the number of predictors, and other characteristics of the data, including its quality (71). The sample size for the individual-level analyses in the present study (i.e., 160 observations per individual) was larger than in similar published studies (72,73) and in line with the larger (rather than smaller) sample sizes evaluated in simulation studies (71),* *which lends confidence to the small number of individual-level algorithms that could be constructed. As the abovementioned class imbalances (i.e., a non-trivial proportion of participants who reported 0% lapses) were likely a greater concern than the sample size in the present study, additional methodological research is needed to better understand* *how sample size and characteristics of the data influence both group- and individual-level algorithm performance.”*

*“We found that high-performing group-level lapse prediction algorithms without and with passively sensed data had variable performance when applied to unseen individuals. Individual-level and hybrid algorithms had improved performance, particularly when incorporating sensor data from a commercially available device for participants with sufficient wear time. However, they could only be constructed for a limited number of individuals due to methodological challenges (discussed in detail below). Hybrid algorithms could be constructed for about twice as many individuals as the individual-level algorithms.”*

*“High-performing group-level lapse prediction algorithms had variable performance when applied to unseen individuals. Individual-level and hybrid algorithms had improved performance, particularly when incorporating sensor data for participants with sufficient wear time. Hybrid algorithms could be constructed for about twice as many individuals as the individual-level algorithms.”*

AUC results (Figures 2 and 5) for group-level analysis could also use a box plot or histogram (similar to Figures 4 and 7) that includes standard deviations to show how close the AUCs from the different models are to one another

*We now include such figures in the Supplementary Materials.*

Introduction could use a few more sentences clarifying how this study is related to identifying best intervention time over best intervention method. This point is included in the Discussion, but it should be made clear earlier on.

*We have now clarified this in the Introduction on p.4:*

*“A key question for JITAI developers is knowing when to intervene for each individual (i.e., identifying decision points and tailoring variables) and what data source(s) to use for reliable inputs. Several approaches– primarily focused on theory-informed selection or predictive modelling – have been developed and tested (summarised in Table 1). Other approaches, which also tend to incorporate the identification of what intervention to provide at moments of need, place a greater emphasis on causal modelling. These approaches include experimental designs (e.g., micro-randomised trials, system identification experiments from control engineering) and formal, dynamical systems modelling (17–20). We do not expand further on such approaches here as it was not our intention in the present study to identify what intervention to provide at moments of predicted need (although see (21,22) for micro-randomised trials in progress within the smoking cessation domain).”*

Discussion could use an interpretation of the ML results – why do specific models work better than others for the various data settings?

*We now provide discussion of the different algorithm types and their performance on p.20:*

*“Overall, Random Forest algorithms outperformed the other algorithm types (i.e., Support Vector Machine, XGBoost, Penalised Logistic Regression); however, it should be noted that the absolute AUC differences were minimal. The Random Forest algorithm has gained popularity due to its flexibility with regards to the functional form of predictor-outcome associations and ability to account for many predictor variables (including higher-order interactions) (61). Some studies show that the Random Forest algorithm outperforms similar algorithms, such as the Support Vector Machine, although results are mixed (61). We therefore caution against placing too much emphasis on the algorithm type in the present study.”*

Discussion could also use an interpretation of the variable importance results – why might certain variables matter more than others?

*We now provide discussion of the variable importance results on p.20:*

*“Using the variable importance method, predictors such as feeling confident in one’s ability to stay quit and feeling motivated to stay quit appeared important for lapse prediction at the group-level. As expected, however, the individual-level analyses indicated that different predictors were important for different individuals. These results can be harnessed by JITAI developers to identify plausible intervention options to try at times of predicted need. However, as highlighted in our previous work (34), we caution against overreliance on the results from the variable importance function and recommend that robustness analyses (e.g., partial dependence plots) and external validation are carried out prior to determining which predictors are most important.”*

Discussion could use more of a description of limitations / confounders and reproducibility in the future. Population applicability – It should be mentioned that current results are limited to individuals from a London-based population and need to be explored in different populations with different environments and different socioeconomic status

*We have added the following limitations in the Discussion on p.23:*

*“Fifth, participants were recruited during different months of the year. It is possible that seasonality might have affected the results. In addition, the current results are limited to London-based individuals who were willing to participate in a research study with high measurement frequency. External algorithm validation in different populations (e.g., individuals with lower socioeconomic position) and settings (e.g., individuals residing in rural or smaller urban environments) is therefore needed. We encourage such explorations, including examinations of seasonality, using our openly available R code in future research.”*

Discussion could use a description of how to translate/productionalize this work – I.e., a few more sentences about how these results might inform future JITAI development.

*We have added the following on p.24:*

*“Taken together, the results from this study highlight several theoretical, methodological, and practical considerations for JITAI developers which, to the best of our knowledge, have not been previously articulated within a single paper. We set out to select a best-performing and feasible algorithm to predict lapses and therefore identify moments of predicted need for intervention by a subsequent JITAI. Although the predictive modelling approach evaluated here shows promise, rather than at this stage acting as a plug-and-play method for the identification of decision points and tailoring variables in future JITAIs, researchers and developers are encouraged to carefully consider: the expected distribution of the outcome variable within individuals over time (and if information is lacking, it may be useful to first conduct a series of descriptive analyses to better understand the phenomenon prior to algorithm training and testing), appropriate algorithm performance metrics to focus on given the distribution of the outcome variable, the feasibility of passive sensing within the target population, ways of managing missing data, and the robustness of different algorithm interpretability methods. With further refinements, the predictive modelling approach deployed in the present study may become a useful off-the-shelf tool for JITAI developers.”*

Minor comments:

GitHub is organized well and provides a good overview of the code for analysis. Commenting in scripts could be improved a bit further, and the README file could include a better overview of the importance of each script.

*We have provided additional information about each script in the README file on GitHub.*

Small grammar error: “Finally, although this study aimed to devise a method to help researchers determine when to intervene, it not address the issue of how to intervene at moments of vulnerability”

*Thank you for spotting - this has now been amended.*

Introduction: “Other approaches to the identification of JITAI decision points and tailoring variables (not expanded on further in this paper)…” This is a long sentence and is hard to follow. Break it down into smaller pieces.

*We have edited this sentence for improved readability:*

*“Other approaches, which also tend to incorporate the identification of intervention options, place a greater emphasis on causal modelling. These approaches include experimental designs (e.g., micro-randomised trials, system identification experiments from control engineering) and formal, dynamical systems modelling (17–20). We do not expand further on such approaches here as it was not our intention in the present study to identify what intervention to provide at times of predicted need (although see (21,22) for micro-randomised trials in progress within the smoking cessation domain).”*

The font size for Figures 2 to 7 could be increased to improve readability

*The font size has now been increased in these figures.*

Reviewer #3

Although evaluating area under ROC curve can be a good metric for model and performance evaluation, it would be preferred to add more metrics which are useful and suitable for clinical analysis applications such as precision-recall and F1-score. It would improve the credibility of the results and help with the comparison and evaluation of AUC for the model performance.

*The thresholds for acceptable accuracy, sensitivity and specificity were pre-registered and grounded in clinical reasoning within the domain of smoking cessation/lapse prevention, with metrics such as the F1 score and precision-recall curve currently lacking validation within this specific domain. We have therefore opted not to report additional performance metrics and mention this as a future direction in the Discussion on p.23:*

*“However, the reliance on specific performance metrics – without considering a wider range of indicators such as the F1 score or the precision-recall curve – constitutes another limitation of the present study which merits attention in future research. The selected performance metrics (i.e., sensitivity, specificity, the area under the receiver operator characteristic curve) cannot be computed when the test set contains only one of the two classes. This was the case for a non-trivial proportion of participants who reported 0% lapses and hence limited the number of participants for whom the algorithms could be constructed. Future research should consider using the Matthews Correlation Coefficient for imbalanced datasets (70), in addition to devising appropriate cut-offs for use in the context of a smoking lapse estimator, which are currently lacking.”*

Also summarising the introduction to some extent might help with the flow and story of the work.

*In response to this comment and similar comments from the other reviewers, we have now substantially rewritten the Introduction (edits in track changes) and included a summary table. In addition, we have clarified the key contribution of our study on p.6:*

*“The key contribution of the present study was therefore to add to the growing literature on the identification of JITAI decision points and tailoring variables by developing and evaluating a series of group-level, individual-level, and hybrid supervised machine learning algorithms to predict lapse risk in smokers attempting to stop, leveraging both EMAs and wearable sensor data. This was with a view to ultimately using the best-performing algorithm within a subsequent JITAI for smoking cessation.”*
