# Supplementary material for: Supervised machine learning to predict smoking lapses from Ecological Momentary Assessments and sensor data: Implications for just-in-time adaptive intervention development
Source: PLOS Digit Health. 2024 Aug 23;3(8):e0000594. doi: 10.1371/journal.pdig.0000594 (PMC11343380; doi:10.1371/journal.pdig.0000594)
Supplement: Attachment — Submitted filename: Reviewer comments 24.06.24.docx [file pdig.0000594.s020.docx]

We thank the editor and the reviewer for their helpful feedback on our manuscript, which we believe have helped strengthen it. Please find our responses to each comment and related amendments below.

**Journal Requirements:**

1. Please provide separate figure files in .tif or .eps format only and remove any figures embedded in your manuscript file. Please also ensure that all files are under our size limit of 10MB.

Separate figure files have now been provided.

2. We have noticed that you have uploaded Supporting Information files, but you have not included a list of legends. Please add a full list of legends for your Supporting Information files after the references list.

A list of legends for the Supporting Information has now been provided after the reference list.

**Additional Editor Comments:**

3. A reviewer provided a useful comment in the last review round: “… if the authors have tried out other machine learning or deep learning algorithms? I felt a simple vanilla ANN with few number of hidden layers seems to be a nice candidate model to evaluate? …” The comment is valid, and I suggest the authors to apply the relevant algorithms to see whether better prediction performance would be obtained, rather than pointing out the issue as a limitation.

Thank you – we have now repeated the analyses using an artificial neural network, with the results presented on p.21:

“As a result of the review process, we subsequently compared the best-performing group-level algorithm (i.e., Random Forest) with a simple Artificial Neural Network. Similar to the other algorithm types, the tidymodels framework of packages (52) was used, setting the engine to nnet (i.e., a single-layer, feed-forward neural net), the mode to classification, and tuning the relevant hyperparameters. The algorithm performance remained similar (but was not superior to) the best-performing group-level algorithm (AUC = 0.853, 95% CI = 0.818-0.887).

After removing participants with 0% lapses, algorithm performance could be computed for 25 participants (25/30; 65.8%). The median AUC was moderate at 0.614 and varied widely across participants (range: 0.509–0.954). Due to non-superiority, we did not proceed with the remaining objectives.”

4. It is also important to note the comment about “how seasonality / time of year might affect the results”. Again, I suggest authors to consider conducting an analysis to address the comment rather than citing it as a limitation.

We have now added a sensitivity analysis with season as an additional predictor, with the results reported on p.21:

“As a result of the review process, we also examined whether an indicator of seasonality (i.e., the time of year when the participants were recruited into the study) influenced algorithm performance. We incorporated the season of the year as a categorical predictor (i.e., summer, autumn, winter, spring). The algorithm type (i.e., Random Forest) and performance (AUC = 0.912, 95% CI = 0.887-0.937) remained similar (but was not superior to) the best-performing group-level algorithm without the inclusion of seasonality.

After removing participants with 0% lapses, algorithm performance could be computed for 25 participants (25/30; 65.8%). The median AUC was moderate at 0.664 and varied widely across participants (range: 0.511–0.981). Due to non-superiority, we did not proceed with the remaining objectives.”

**Reviewer #4:**

This study (n=38 smokers attempting to quit; 7% reported a lapse) examined the use of EMA and Fitbit data collected over 10-days to predict smoking lapse (and in sensitivity analyses: craving dichotomously coded). Individual and hybrid (combination of individual and group-level algorithms) models performed better than group-level models, and sensor data added predictive info above EMA data alone. However, the addition of sensor data needs to be considered against feasibility because fewer individuals provided both types of data.

The revision is responsive to comments: Introduction was re-organized and includes a summary table of prior relevant research; Discussion addresses limitations regarding the models tested (e.g., no deep learning models), limited model performance metrics examined, and limitations to generalizability of results; and the need for caution in interpreting results when sample size is low and outcome/data are sparse.

Thank you for the helpful feedback on our manuscript.

Some minor additional comments could be considered:

1. Methods section mentions potential confounding factors affecting heart rate, which were assessed in EMA (e.g., caffeine use, physical activity). However, the way in which these possible confounds were taken into account in the analyses when examining heart rate as a predictor of lapse is unclear, and could be addressed (e.g., in supplementary materials) since the confounds might contribute an important source of error.

We have now added a sensitivity analysis in which timepoints with potential confounders were removed, reported on p.22 and in the Supporting Information, Figure S12-S13:

“Additional unplanned sensitivity analyses with timepoints with potential confounders removed (i.e., when responses indicated walking/exercising, caffeine intake, nicotine use) are reported in the Supporting Information, Figure S12-S13. The results remained largely robust; however, individual- and hybrid-level algorithms could be constructed for fewer participants (i.e., 9 and 17 participants, respectively).”

We have also added the following to the Discussion on p.25:

“We found initial evidence that features relating to heart rate (e.g., change in slope) may be important predictors of lapses; however, further research is needed to replicate and extend these early results, drawing on techniques from the signal processing literature to filter out noise pertaining to, for example, physical activity or nicotine use (33). Sensitivity analyses were conducted with segments where participants indicated that they were walking or exercising, or consuming caffeine or nicotine were removed. However, further research is required to better understand what aspects of heart rate are useful for lapse prediction, under what conditions, and why – such work has been referred to as digital biomarker discovery (70).”

2. Page 10: “participants with insufficient sensor wear time were removed from the analytical sample, defined as >20% adherence…” The phrasing of this statement seems to suggest that the definition refers to “insufficient” wear time. It might be clearer to rephrase this to, e.g.: “participants with sufficient sensor wear time were defined as having >20% adherence…”

This has now been amended as suggested on p.10:

“Finally, participants with insufficient sensor wear time were removed from the analytical sample. Participants with sufficient wear time were defined as having ≥20% adherence on ≥5 of the 10 study days.”
