# Supplementary material for: Supervised machine learning to predict smoking lapses from Ecological Momentary Assessments and sensor data: Implications for just-in-time adaptive intervention development
Source: PLOS Digit Health. 2024 Aug 23;3(8):e0000594. doi: 10.1371/journal.pdig.0000594 (PMC11343380; doi:10.1371/journal.pdig.0000594)
Supplement: Attachment — Submitted filename: Reviewer comments 18.07.24.docx [file pdig.0000594.s021.docx]

We thank the reviewer for their helpful feedback on our manuscript. Please find our responses to the comments below.

1. Grammatical problems. The last sentence of the first paragraph of "Data analysis," "(i.e., cravings at t0 was used to predict lapse incidence at t1)." is inconsistent. Please revise the sentence and confirm the whole text for no grammatical or spelling problems.

This sentence has now been amended:

“As we aimed to *predict* rather than *detect* lapses, the input variables were used to predict the target variable (i.e., lapse incidence) at the subsequent time point (i.e., the next hour).”

We have carefully checked the manuscript for additional grammatical or spelling problems.

2. The first sentence in the "Model training and testing" of the "Data analysis" section： "Model training and testing was performed through k-fold cross-validation (55), with k set to 10. For each iteration (or fold), models were trained on 80% of the data and tested on the remaining 20% of the data." The expression of the k-fold cross-validation used is unclear and ambiguous. The current statement does not meet the definition of k-fold cross-validation. If a stratified k-fold method was used, please indicate the basis or reasons for such stratification.

Thank you for spotting this. We have now clarified the procedure as follows:

“First, we created train-test splits of the datasets, with 80% of the data used for training and 20% kept for testing (i.e., an unseen ‘holdout sample’). Next, the models were trained on the train splits using k-fold cross-validation (55), with k set to 10. The models were subsequently tested on the unseen hold-out samples (i.e., the test splits) and performance metrics were calculated.”

3. Table 2 shows that the “Welch Two Sample t-test” and “Pearson's Chi-squared test” were employed. Please supplement the statistical methods used in the data analysis and the purpose.

We have now added details about the t-tests and Chi-squared tests in the data analysis section on p.10:

“All analyses were conducted using R Statistical Software (52). First, descriptive statistics (e.g., the mean and standard deviation) were calculated to describe the characteristics of the overall sample, the analytic sample, and those excluded due to not meeting the adherence cut-offs. T-tests and Chi-squared tests, as appropriate, were used to compare the characteristics of the analytic sample with those excluded.”
